# Inactivation of DNase1L2 and DNase2 in keratinocytes suppresses DNA degradation during epidermal cornification and results in constitutive parakeratosis

**DOI:** 10.1038/s41598-017-06652-8

**Published:** 2017-07-25

**Authors:** Heinz Fischer, Maria Buchberger, Markus Napirei, Erwin Tschachler, Leopold Eckhart

**Affiliations:** 1 Research Division of Biology and Pathobiology of the Skin, Department of Dermatology, Medical University of Vienna, 1090 Vienna, Austria; 2 Department of Anatomy and Molecular Embryology, Medical Faculty, Ruhr-University Bochum, 44801 Bochum, Germany; 3 Unit of Pathology of Laboratory Animals, University of Veterinary Medicine, 1210 Vienna, Austria

## Abstract

The stratum corneum of the epidermis constitutes the mammalian skin barrier to the environment. It is formed by cornification of keratinocytes, a process which involves the removal of nuclear DNA. Here, we investigated the mechanism of cornification-associated DNA degradation by generating mouse models deficient of candidate DNA-degrading enzymes and characterizing their epidermal phenotypes. In contrast to *Dnase1l2* ^−/−^ mice and keratinocyte-specific DNase2 knockout mice (*Dnase2* ^*Δep*^), *Dnase1l2* ^−/−^ *Dnase2* ^*Δep*^ mice aberrantly retained nuclear DNA in the stratum corneum, a phenomenon commonly referred to as parakeratosis. The DNA within DNase1L2/DNase2-deficient corneocytes was partially degraded in a DNase1-independent manner. Isolation of corneocytes, i.e. the cornified cell components of the stratum corneum, and labelling of DNA demonstrated that corneocytes of *Dnase1l2* ^−/−^ *Dnase2* ^*Δep*^ mice contained DNA in a nucleus-shaped compartment that also contained nucleosomal histones but lacked the nuclear intermediate filament protein lamin A/C. Parakeratosis was not associated with altered corneocyte resistance to mechanical stress, changes in transepidermal water loss, or inflammatory infiltrates in *Dnase1l2* ^−/−^ *Dnase2* ^*Δep*^ mice. The results of this study suggest that cornification of epidermal keratinocytes depends on the cooperation of DNase1L2 and DNase2 and indicate that parakeratosis *per se* does not suffice to cause skin pathologies.

## Introduction

Epidermal keratinocytes differentiate from proliferating cells attached to the basement membrane to metabolically inert but mechanically resilient corneocytes within the outermost layer of the body, the stratum corneum. The terminal step of the keratinocyte differentiation program is cornification, a unique form of programmed cell death which involves the coordinated cross-linking of structural proteins via transglutamination and the breakdown of cell organelles by incompletely understood mechanisms^1, 2^. The nucleus is degraded during normal cornification resulting in corneocytes that are homogeneously filled with keratin (orthokeratosis). However, in lesions of psoriasis, atopic dermatitis, and other skin diseases the nucleus is retained in corneocytes (parakeratosis)^3, 4^. In mice and human *in vitro* skin models, various treatments and gene modifications disturb terminal differentiation of keratinocytes and manifest in parakeratosis, but mechanistic insights into the development of parakeratosis have remained scarce^5–9^.

Nuclear DNA is degraded during orthokeratotic cornification of keratinocytes in the epidermis and in skin appendages^10, 11^ whereas DNA degradation does not occur or remains incomplete during parakeratotic cornification of keratinocytes. In human *in vitro* skin models, the keratinocyte-specific enzyme DNase1L2 is required for nuclear DNA degradation during stratum corneum formation^12^. DNase1L2 also counteracts the formation of bacterial biofilms *in vitro* ^13^. Deletion of DNase1L2 in mice blocked DNA degradation in hair, nails, filiform papillae of the tongue, in the epithelia of the oral cavity, the esophagus and the prestomach^14^, as well as in the cuticle of the inner root sheeth of hair follicles^15^. However, the stratum corneum of DNase1L2 knockout mice is orthokeratotic, suggesting that DNase1L2 is not essential for DNA degradation during cornification of interfollicular keratinocytes in mice^14^. In contrast to the results of DNase1L2 knockdown, the suppression of the lysosomal DNase2 abrogated acid DNase activity in human skin equivalents but did not lead to parakeratosis^16^. Mice in which *Dnase2* was inactivated by the Cre-loxP system in keratin K14-positive cells and their progeny cells (*Dnase2* ^*Δep*^), including epidermal keratinocytes and sebocytes, displayed incomplete DNA degradation during holocrine secretion of sebum but did not result in parakeratosis in the interfollicular epidermis^15^.

Here, we investigated whether DNase1L2 and DNase2 act together in mouse stratum corneum formation. We show that *Dnase1l2* ^−/−^ *Dnase2* ^*∆ep*^ mice develop parakeratosis and suggest that DNase1L2, DNase2, and at least one more DNA-degrading enzyme degrade DNA during cornification of keratinocytes.

## Material and Methods

### Mice

The generation of *Dnase1l2* ^−/−^^14^, *Dnase1* ^−/−^^17, 18^, *Dnase2* ^*∆ep*^, *Dnase1l2* ^−/−^ *Dnase2* ^*∆ep*^, and *Dnase1* ^−/−^ *Dnase1l2* ^−/−^ *Dnase2* ^*∆ep*^ ^15^ mice was reported previously. *Dnase1* knockout and *Dnase1l2* knockout mice were derived from breeding colonies described previously^14, 17^. In *Dnase2* ^*∆ep*^, also referred to as *Dnase2* ^*f*/*f*^ *K14-Cre* mice, essential exons of the *Dnase2a* gene are flanked by loxP sites (*Dnase2* ^*f*/*f*^)^19^ and the *Cre* transgene is expressed under the control of the *Krt14* promoter. Thus, *Dnase2* is deleted in epidermal keratinocytes which express *Krt14* in the basal layer before moving towards the skin surface. All mice were maintained according to the animal welfare guidelines of the Medical University of Vienna, Austria. Experiments on live animals were approved by the Federal Ministry of Economy and Research (BMWF), Austria, under the approval numbers: BMWF-66.009/0213-II/2008; BMWF-66.009/0231-II/3b/2012.

Trans-epidermal water loss (TEWL) was measured on the ears and footpads with a Tewameter from Courage and Khazaka, model TM300 (Cologne, Germany).

### Preparation of tissue samples for histology

Tissues were prepared immediately after sacrificing mice by cervical dislocation. Skin samples were fixed in phosphate-buffered 7.5% formaldehyde for 24 hours and subsequently embedded in paraffin.

### TUNEL

The terminal deoxynucleotidyl transferase dUTP nick end labeling (TUNEL) assay (Roche Diagnostics GmbH, Vienna, Austria) was performed to label 3′-OH ends of DNA *in situ*, according to a published protocol^20^. The sections were also subjected to DNA labeling with Hoechst 33258 (Sigma Aldrich, St. Louis, MO). The results were documented using an Olympus AX 70 microscope (Hamburg, Germany), a Spot RT3 slider camera (SPOT Imaging Solutions, Sterling Heights, MI), and the imaging software Metamorph (Visitron Systems, Puchheim, Germany).

### Histological investigations and immunofluorescence labeling

Tissue sections were stained with hematoxylin and eosin (H&E). Mast cells were stained with 1% toluidine blue^21^. T cells were labeled by with rabbit anti-CD3 (1:800, Glostrup, Denmark). Other immunolabelings were done with rabbit anti-histone H3 antiserum (1:50, Cell Signaling Technology, Danvers, MA) and mouse anti-lamin A/C (1:200, Cell Signaling Technology, Danvers, MA). Secondary antibodies conjugated to Alexa Fluor 546 fluorescent dye (Molecular Probes, Leiden, Netherlands) were used at a dilution of 1:500. When the primary antibodies were replaced by unrelated control antibodies, the fluorescence signals were abolished, confirming the specificity of the immunolabelings.

### Preparation and investigation of corneocytes

Corneocytes were prepared by tape stripping of the skin surface using D-squames^®^ (Cuderm, Dallas, TX). The corneocytes attached to the tape were subjected to DNA-labeling and visualized by fluorescence microscopy and bright-field microscopy with phase contrast. The corneocyte stress test was performed as described previously^22^ with modifications. In brief, the epidermis of ear skin was separated from the dermis by treatment with dispase and subsequently incubated in PBS containing 2% SDS at a temperature of 95 °C for 10 min. The remaining corneocytes were centrifuged and resuspended in the same buffer at a density of 10^6^/ml. The corneocyte suspensions were sonicated in a sonicator (Hielscher UIS250L, Teltow, Germany). Aliquots were removed every 10 seconds, and intact corneocytes were counted with a counting chamber under the microscope. At least 3 biological replicates were measured. Differences were evaluated with the two-sided t-test. *P*-values greater than 0.05 were considered not significant.

### Data availability statement

The datasets generated and analysed during the current study are available from the corresponding author on reasonable request.

## Results

### Co-deletion of DNase1L2 and DNase2 causes epidermal parakeratosis

**Figure 1:**
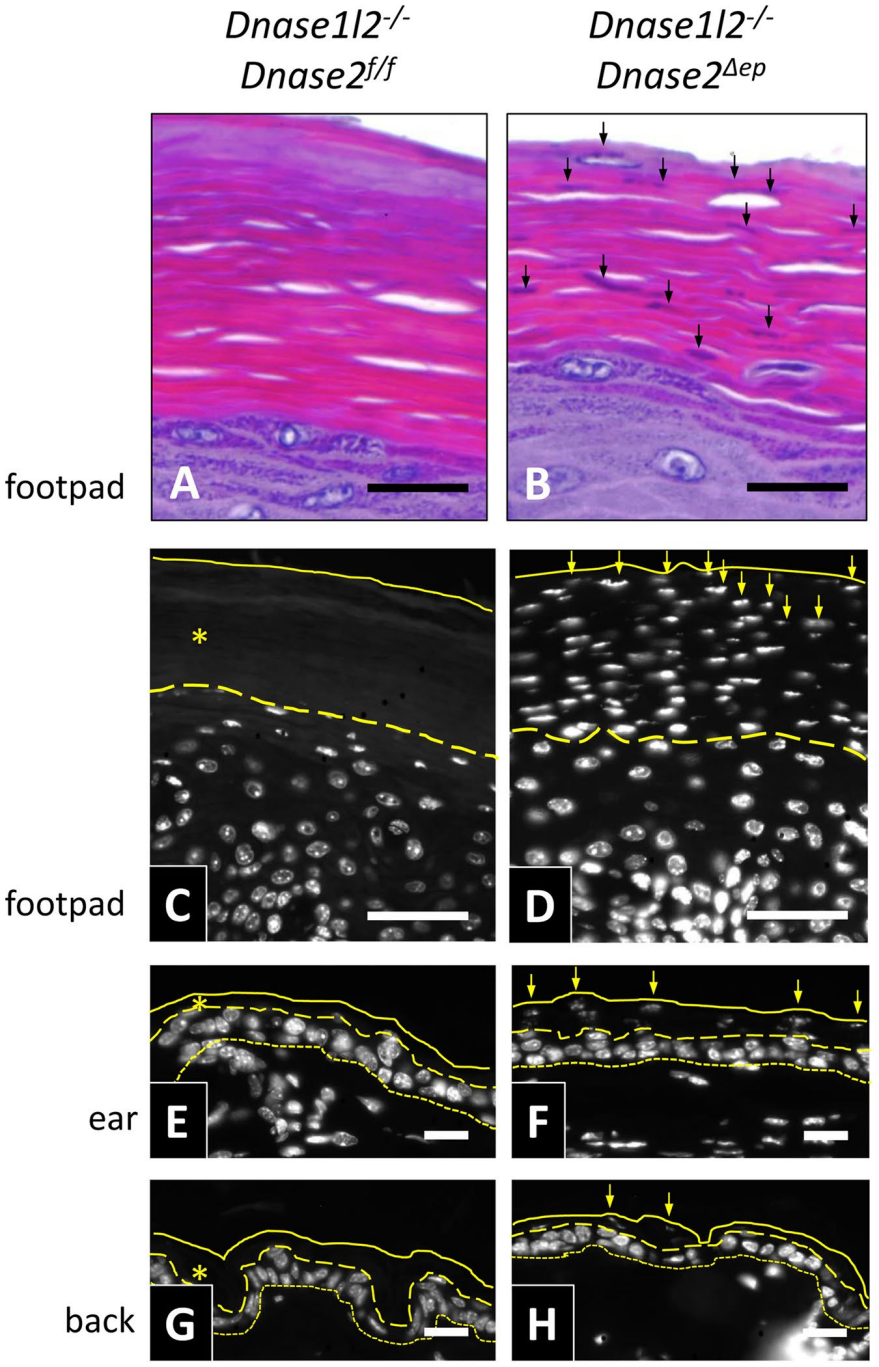
DNase1L2/DNase2 double knockout causes parakeratosis. Thin section of the skin from *Dnase1l2* ^−/−^ (**A**,**C**,**E**,**G**) and *Dnase1l2* ^−/−^ *Dnase2* ^*Δep*^ (**B**,**D**,**F**,**H**) mice were stained with hematoxylin and eosin (**H** and **E**) (**A**,**B**) and the DNA-specific dye Hoechst 33258 (**C**–**H**). The skin was prepared from the footpads (**A**,**D**), ears (**E**,**F**), and back (**G**,**H**). Continuous and dashes lines indicate the outer and the inner border of the stratum corneum, respectively. The basal layer of the epidermis is indicated by dotted lines. Arrows indicate nuclear remnants detected in terminally differentiated corneocytes of *Dnase2* ^*∆ep*^ mice. The data are representative of at least 3 mice per genotype. Scale bars, 20 µm.

H&E staining and DNA labelling with Hoechst 33258 dye showed that, in agreement with previous reports^14, 15^, wild-type mice as well as *Dnase1l2* ^−/−^, and *Dnase2* ^*f*/*f*^ *K14-Cre* mice (here termed *Dnase2* ^*∆ep*^ mice with *∆ep* short for deletion in epithelial cells derived from K14-positive precursors) had an orthokeratotic stratum corneum (Fig. 1A,C,E,G, and data not shown). Mice lacking both DNase1L2 and DNase2 in epidermal keratinocytes, were generated by crossing *Dnase1l2* ^−/−^ mice with *Dnase2* ^*Δep*^ mice. H&E staining showed distinct nuclear remnants in the stratum corneum (parakeratosis) on the soles and footpads of *Dnase1l2* ^−/−^ *Dnase2* ^*Δep*^ mice (Fig. 1B). No nuclear remnants were detected within the stratum corneum on the ear and back skin after H&E staining (Suppl. Fig. S1). However, DNA labelling with Hoechst 33258 dye revealed the presence of nuclear remnants within the stratum corneum also of these body sites of *Dnase1l2* ^−/−^ *Dnase2* ^*Δep*^ mice (Fig. 1D,F,H). In line with the H&E stainings, the aberrant retention of DNA was most prominent in the epidermis of the footpads (Fig. 1D).

### DNA in parakeratotic stratum corneum of *Dnase1l2*^−/−^*Dnase2*^*Δep*^ mice is fragmented

**Figure 2:**
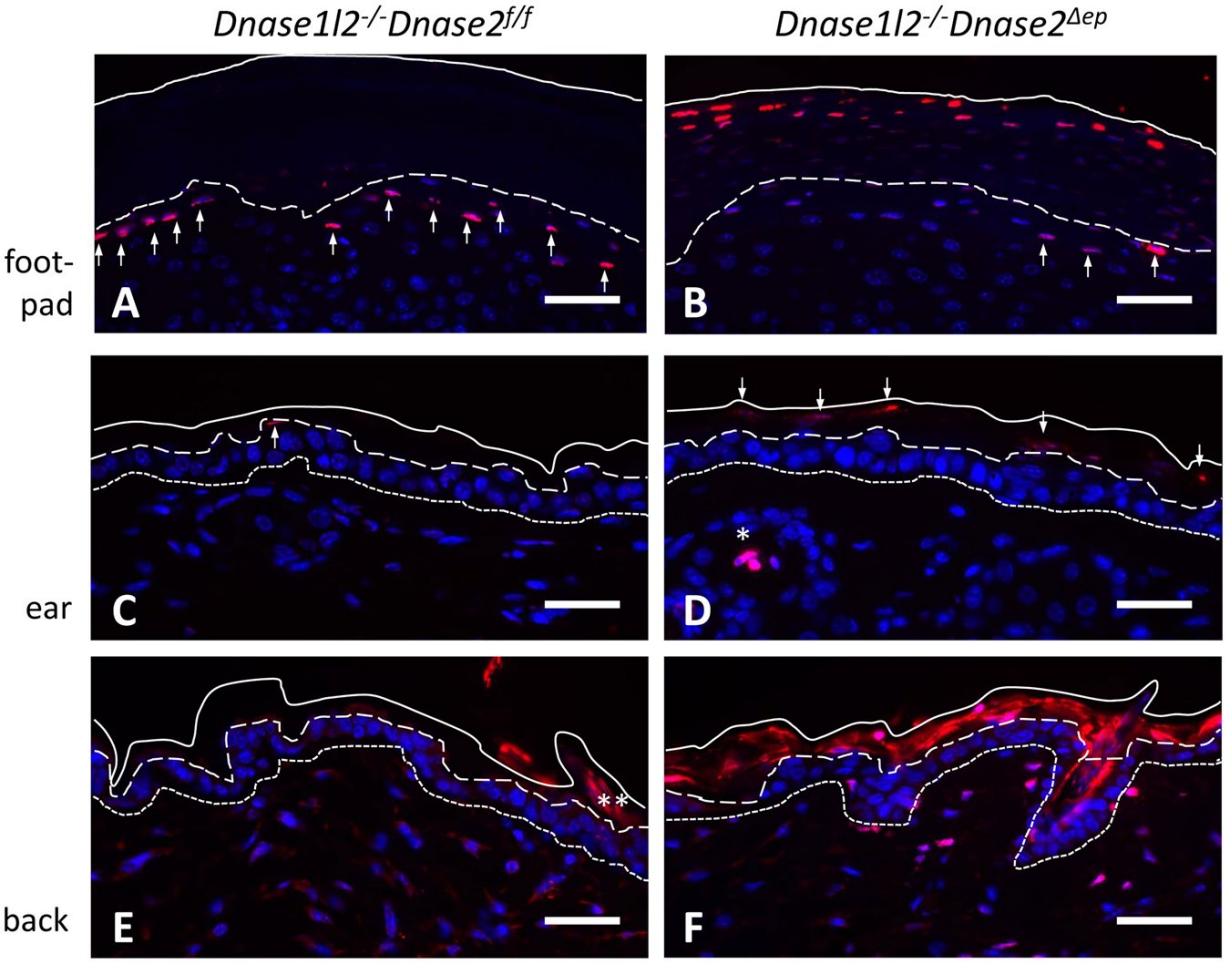
DNA in DNase1L2/DNase2-deficient corneocytes is partially degraded. Thin section of the skin on the footpads (**A**,**B**), ears (**C**,**D**), and back (**E**,**F**) of *Dnase1l2* ^−/−^ (**A**,**C**,**E**) and *Dnase1l2* ^−/−^ *Dnase2* ^*Δep*^ (**B**,**D**,**F**) mice were subjected to TUNEL labeling and counter-stained with the DNA-specific dye Hoechst 33258. Continuous and dashes lines indicate the outer and the inner border of the stratum corneum, respectively. The basal layer of the epidermis is indicated by dotted lines. The data are representative of at least 3 mice per genotype. Scale bars, 20 µm.

The TUNEL assay showed that parakeratotic stratum corneum of *Dnase1l2* ^−/−^ *Dnase2* ^*Δep*^ mice contained free 3′-OH ends, indicating that the retained DNA was at least partially fragmented (Fig. 2). As DNase1 is a candidate enzyme for generating these DNA ends, we generated *DNase1* ^−/−^ *Dnase1l2* ^−/−^ *Dnase2* ^*Δep*^ mice. Deletion of DNase1 alone did not result in parakeratosis, and deletion of DNase1 in addition to the deletion of DNase1L2 and DNase2 did not abrogate the TUNEL-positivity of parakeratotic corneocytes (Suppl. Fig. S2 and data not shown). These results argue against a critical role of DNase1 in cornification. Thus, the partial degradation of DNA in the stratum corneum of *Dnase1l2* ^−/−^ *Dnase2* ^*Δep*^ mice is due to an as yet unknown mechanism.

**Figure 3:**
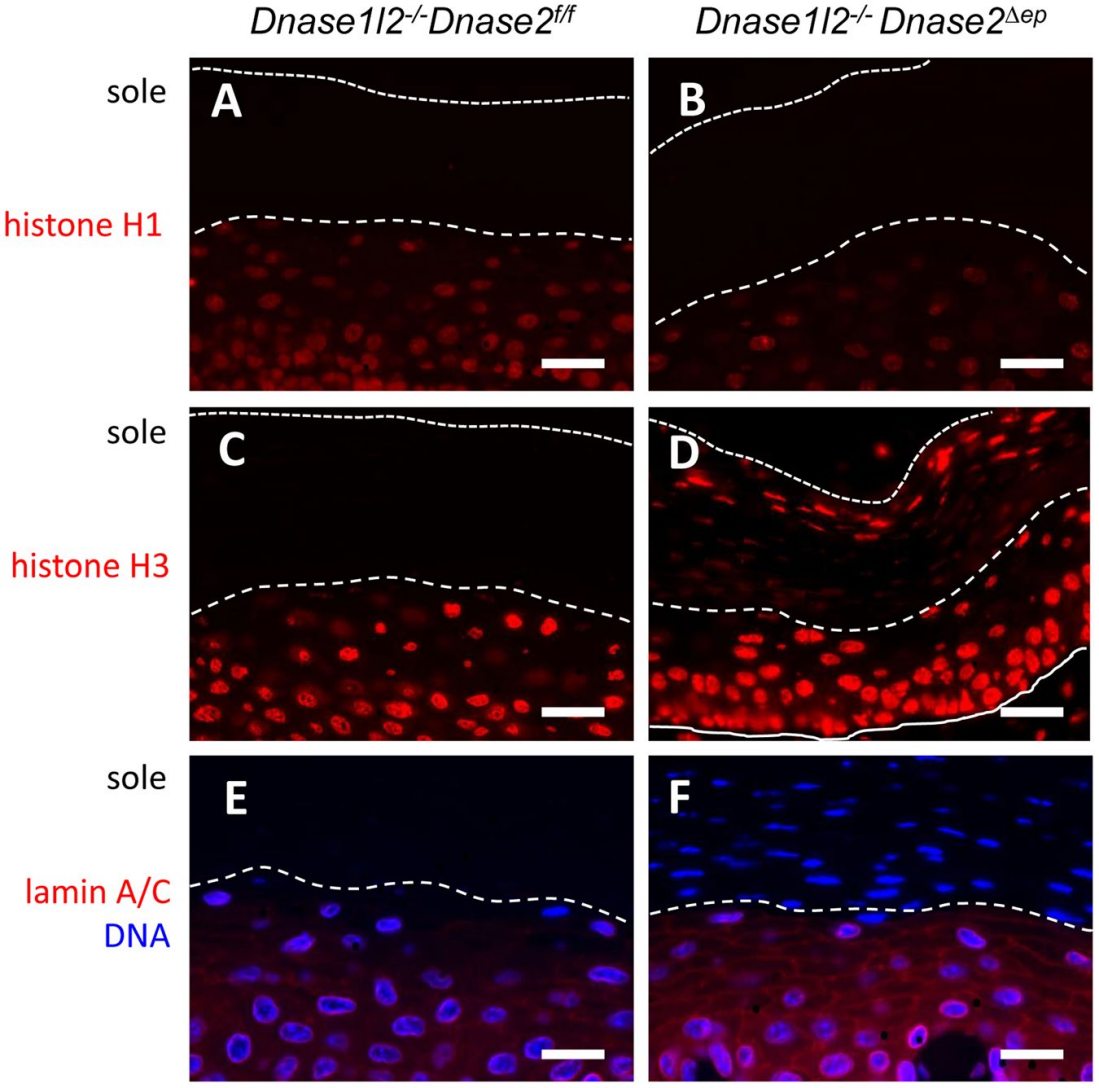
Immunofluorescence labeling of histones and lamin. Thin section of the skin on the soles of *Dnase1l2* ^−/−^ (**A**,**C**,**E**) and *Dnase1l2* ^−/−^ *Dnase2* ^*Δep*^ mice (**B**,**D**,**F**) were immunolabeled with antibodies against histone H1 (**A**,**B**), H3 (**C**, **D**), and lamin A/C (**E**,**F**). The sections were counter-stained with the DNA-specific dye Hoechst 33258. Dotted and dashes lines indicate the outer and the inner border of the stratum corneum, respectively. The data are representative of at least 3 mice per genotype. Scale bars, 20 µm.

Immunofluorescence analysis of wild-type mice demonstrated that normal cornification involves the complete removal of histones H1 and H3 (Fig. 3). By contrast, in DNase2/DNase1L2-deficient keratinocytes histone H3, which is part of nucleosomes, was retained during cornification (Fig. 3C,D) while the internucleosomal histone H1 (Fig. 3A,B) and the nuclear intermediate filament protein, lamin A/C (Fig. 3E,F) behaved like in wild-type mice. These data suggest that DNA degradation is a necessary prerequisite for enabling degradation of histone H3 during normal cornification whereas nuclear proteins that are not enwrapped by DNA are degraded independently of DNase activities.

### DNase1L2/DNase2-deficient corneocytes retain DNA in a nucleus-shaped compartment, display normal resistance to stress and establish a functional skin barrier

**Figure 4:**
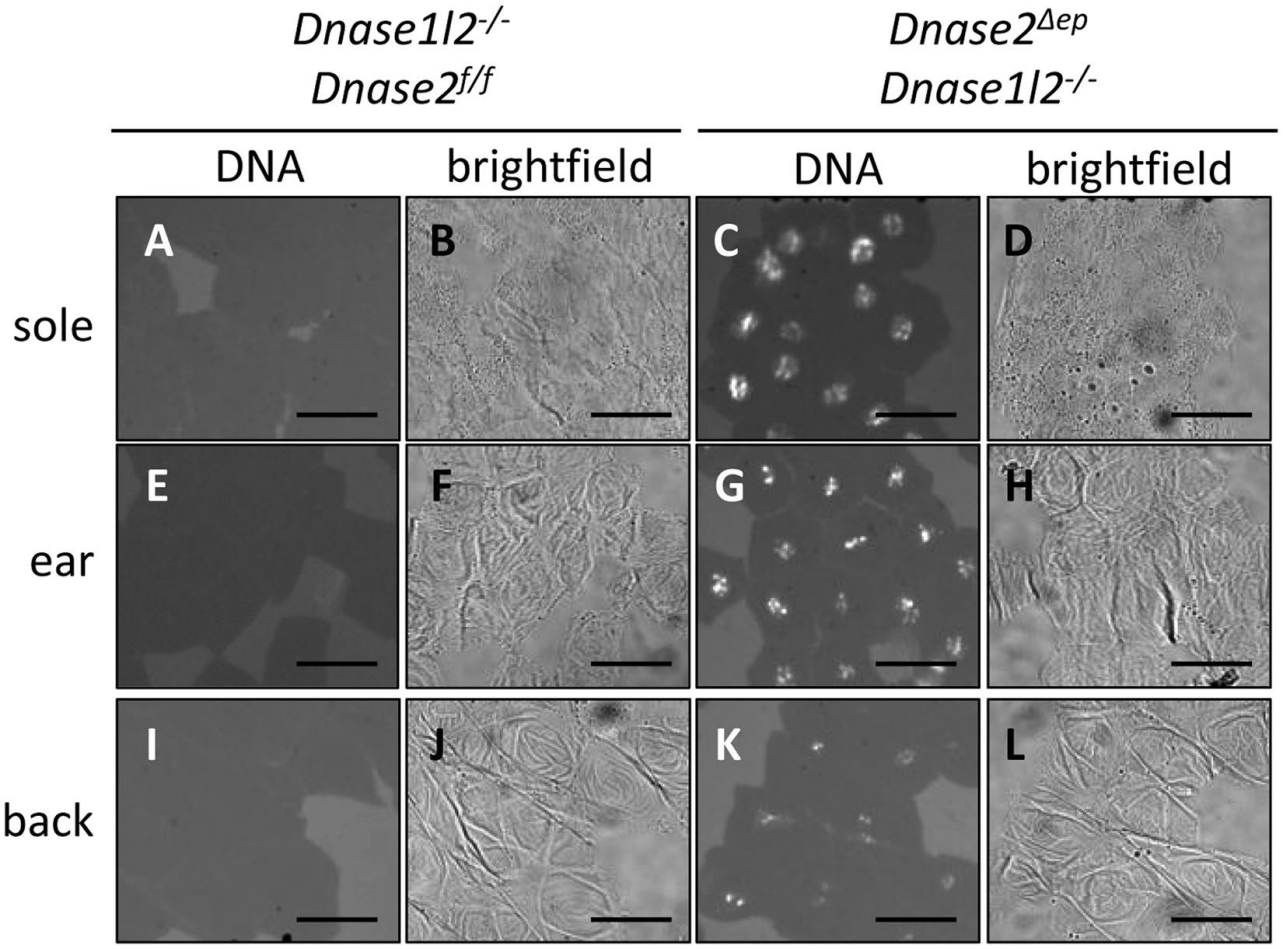
Isolated corneocytes of DNase1L2/DNase2-deficient mice retain DNA in a nucleus-shaped compartment. Corneocytes were isolated from the soles (**A–D**), ears (**E–H**), and back (**I**–**L**) of wild-type (**A**,**B**,**E,F**,**I**,**J**) and *Dnase1l2* ^−/−^ *Dnase2* ^*Δep*^ (**C**,**D**,**G,H**,**K**,**L**) mice. The corneocytes were incubated with DNA-specific dye Hoechst 33258 and fluorescence images in which white signals indicate the labeling (left panels) as well as bright-field images under phase contrast (right panels) were recorded. Scale bars, 20 µm.

Next, we isolated corneocytes from *Dnase1l2* ^−/−^ *Dnase2* ^*Δep*^ and wild-type mice, and incubated them with DNA-specific dye. Corneocytes from *Dnase1l2* ^−/−^ *Dnase2* ^*Δep*^ mice consistently contained DNA that appeared to be concentrated in nucleus-shaped compartments (Fig. 4). Bright field microscopy revealed that corneocytes isolated from *Dnase1l2* ^−/−^ *Dnase2* ^*Δep*^ mice remained intact during the isolation procedure and had morphologies comparable to those from wild-type mice (Fig. 4). Exposure to ultrasound also resulted in comparable decay of wild-type and DNase1L2/DNase2 double knockout corneocytes (Suppl. Fig. S3), indicating that the retention of DNA did not increase the sensitivity of corneocytes to mechanical stress.

Measurement of the trans-epidermal water loss (TEWL) in wild-type and *Dnase1l2* ^−/−^ *Dnase2* ^*Δep*^ mice did not reveal significant differences between the two genotypes (Suppl. Fig. S4). *In situ* immunostaining for inflammatory cells in the skin (Suppl. Fig. S5) showed no significant difference between *Dnase1l2* ^−/−^ *Dnase2* ^*Δep*^ and wild-type mice (Suppl. Fig. S5). These results suggest that parakeratosis due to DNase2/DNase1L2-deficiency of keratinocytes does neither impair the barrier function of the stratum corneum nor trigger an inflammatory reaction in the skin.

## Discussion

**Figure 5:**
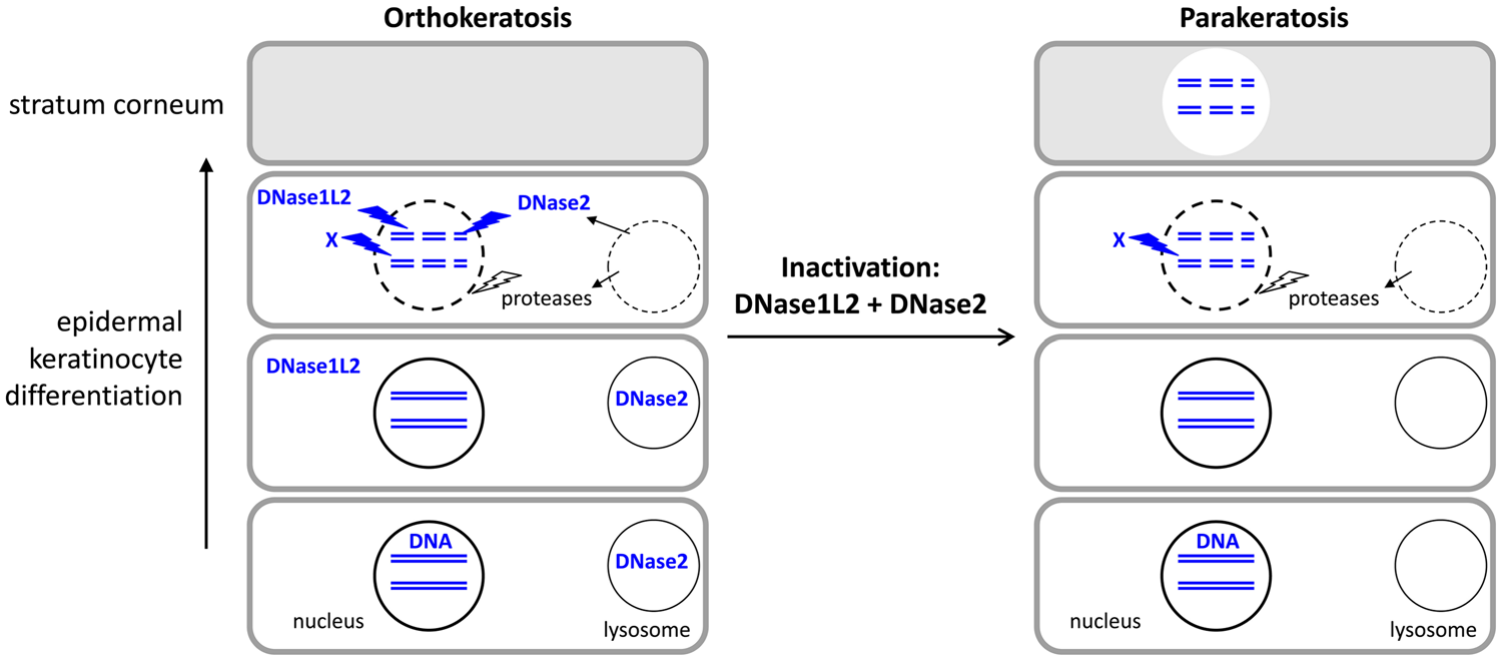
Schematic model of DNA degradation steps during stratum corneum formation. This schematic drawing summarizes steps of the DNA degradation process during cornification of interfollicular keratinocytes, as inferred from the results of this study. X, unidentified neutral DNase.

In this study both DNase1L2 and DNase2 were identified as enzymes that participate in the degradation of DNA during cornification in the interfollicular epidermis of mice. Previously, we have generated DNase1L2 knockout mice which display aberrant retention of DNA in the corneocytes of hair fibers, inner root sheaths of hair follicles, nails, tail scales, and filiform papillae of the tongue^14, 15^. We have also generated *Dnase2* ^*Δep*^ mice which show aberrant retention of DNA in sebocytes and in the isthmus of the hair follicle^12^. But none of these two mouse strains had parakeratosis in the interfollicular epidermis of the back and ear skin nor on the plantar skin. The absence of epidermal parakeratosis in DNase1L2 knockout mice was surprising because suppression of DNase1L2 alone sufficed to cause parakeratosis in an *in vitro* model of human skin^12^. The results of the present study suggest that DNase1L2 contributes to DNA degradation also during stratum corneum formation in the mouse. However, only when both DNase1L2 and DNase2 are inactivated, DNA degradation in corneocytes remains incomplete. Thus, murine cornification-associated DNA degradation appears to be a multi-step process in which the abrogation of either DNase1L2 or DNase2 activities alone, but not the suppression of both enzymes, can be compensated (Fig. 5).

Our finding that DNA in the parakeratotic stratum corneum of *Dnase1l2* ^−/−^ *Dnase2* ^*Δep*^ mice is TUNEL-positive suggests that it is partly degraded. We could exclude that this degradation depends on DNase1, which is also expressed in differentiating keratinocytes^23^. Other candidate contributors to cornification-associated DNA fragmentation include, but are not limited to, caspase-activated DNase (CAD), also known as DNA fragmentation factor B^24^ and the exonuclease TREX2^10, 25, 26^.

Like in suppression of DNA breakdown during programmed cell death of hair and nail keratinocytes^14^ and sebocytes^15^, the blockade of DNA degradation by deletion of DNase1L2 and DNase2 prevented the complete removal of histone H3, a component of nucleosomes, whereas the inter-nucleosomal histone H1 and a nuclear protein without DNA interaction, lamin A/C, were degraded. Of note, the immunoreactivity of H3 was reduced in the lower but not in the upper layers of the stratum corneum in *Dnase1l2* ^−/−^ *Dnase2* ^*Δep*^ mice (Fig. 3D), suggesting that the epitope targeted by the anti-H3 antibody was transiently masked by a cornification-related mechanism. We propose that DNA degradation via differential contributions of DNase1L2 and DNase2 exposes histone H3, which is otherwise physically protected by DNA, to the action of proteases in terminally differentiating keratinocytes that give rise to three distinct products, i.e. hard skin appendages^14^, sebum^15^, and the stratum corneum (this study). Interestingly, a previous report has suggested that AKT1 signaling and degradation of lamin A/C are essential in the cascade of events that initiate DNA degradation during cornification^27^. Our data suggest that the breakdown of lamin is not sufficient to allow for the breakdown of the nucleus.

The persistence of DNA in keratinocytes undergoing programmed cell death was not associated with an inflammatory reaction in the skin of *Dnase1l2* ^−/−^ *Dnase2* ^*Δep*^ mice. This suggests that the aberrantly retained DNA did not reach or activate sensors of mislocalized DNA, such as TLR9, AIM2, and cGAS^28–30^. The DNA appeared to be concentrated in the center of corneocytes of *Dnase1l2* ^−/−^ *Dnase2* ^*Δep*^ mice (Fig. 4) whereas the periphery of these corneocytes appeared to devoid of DNA, perhaps indicating a physical or chemical mechanism for DNA retention in the position of the nucleus or a DNase1L2 and DNase2-independent mechanism for DNA-degradation in the cytosolic part of corneocytes. The cornified envelope represents another likely barrier against the leakage of DNA out of parakeratotic corneocytes which is a prerequisite for reaching receptors for DNA in living cells of the skin. Alternatively, it is conceivable that DNA in cornifying DNase1L2/DNase2 double knockout keratinocytes is partly taken up by other cells without activating inflammation.

Parakeratosis is an important phenomenon in the diagnosis of many skin diseases^3^, but it has remained unclear whether the retention of nuclear constituents *per se* may contribute to the pathogenesis of skin diseases^31–33^. The results of this study demonstrate that the presence of nuclear DNA in association with histones in corneocytes does not suffice to cause pathological changes. It remains to be investigated whether aberrantly retained DNA in cornifying mouse keratinocytes has a pro-inflammatory effect when the epidermis is exposed to different types of stress. Finally, it is possible that the mouse model does not replicate all relevant processes active in human skin. The expression of DNase1L2 is downregulated in parakeratotic skin lesions of psoriasis patients^12^ and DNA degradation activity at neutral pH, corresponding to DNase1 family members, is reduced in psoriatic scales^34^. By contrast, DNA-degradation activity at acidic pH, corresponding to DNase2, is present in extracts from psoriatic scales^34^, indicating that DNase2 is expressed in sufficent amounts in psoriatic skin. However, it is currently unknown whether DNase2 is proteolytically activated in aberrantly differentiated epidermis^35^ or whether the elevated pH in psoriatic skin, as compared to normal skin^36^, reduces DNase2 activity. Further studies in human skin are necessary to determine whether suppression of DNase1L2 and DNase2 activities contribute to parakeratosis in human skin diseases and to evaluate the response to incomplete DNA degradation in cornifying human keratinocytes.

Our results establish the concept that multiple DNases, including DNase1L2 and DNase2, participate in cornification-associated degradation of nuclear DNA in the interfollicular epidermis, and provide a mechanistic basis for studying the roles of parakeratosis in clinical studies.

## Electronic supplementary material

Supplementary Information
