# Supplementary material for: Inactivation of DNase1L2 and DNase2 in keratinocytes suppresses DNA degradation during epidermal cornification and results in constitutive parakeratosis
Source: Sci Rep. 2017 Jul 25;7:6433. doi: 10.1038/s41598-017-06652-8 (PMC5527052; doi:10.1038/s41598-017-06652-8)
Supplement: Supplementary file 1 — Supplementary Information [file 41598_2017_6652_MOESM1_ESM.pdf]

Supplementary Data

Inactivation of DNase1L2 and DNase2 in keratinocytes suppresses DNA degradation during epidermal cornification and results in constitutive parakeratosis

Heinz Fischer, Maria Buchberger, Markus Napirei, Erwin Tschachler, Leopold Eckhart

Content

Supplementary Figures S1-S5

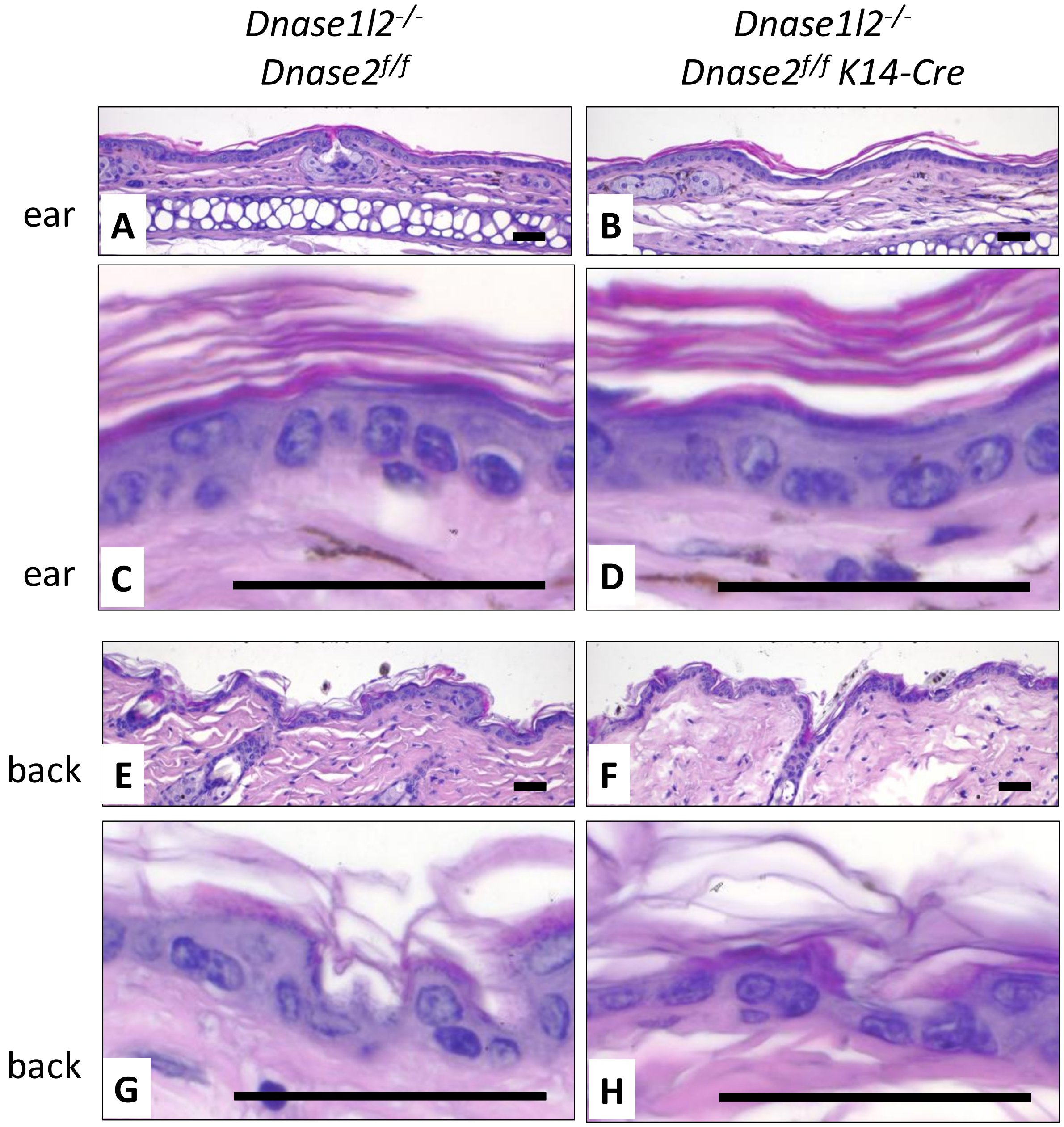

Suppl. Fig. S1. Hematoxylin and eosin (H&E) staining of the skin on the ears and back. DNase1L2 knockout (**A**, **C**, **E**, **G**) and DNase1L2/DNase2 double knockout (**B**, **D**, **F**, **H**) were stained with H&E. Note that, in contrast to the Hoechst labeling of the same skin sites (Fig. 1F, H), H&E staining did not reveal nuclear remnants, suggesting that H&E staining is less sensitive for parakeratosis than Hoechst labeling. The data are representative of at least 3 mice per genotype. Scales bars, 20 µm.

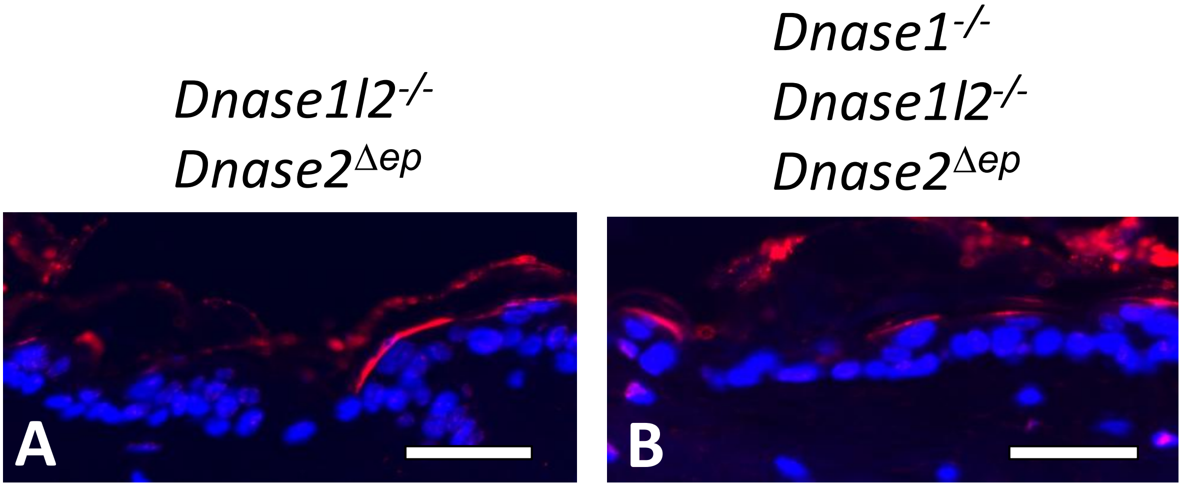

Suppl. Fig. S2. The DNA in the parakeratotic stratum corneum of DNase1/DNase1L2/DNase2-triple-knockout mice is partially degraded. Skin of DNase1L2/DNase2 double knockout (**A**) and DNase1/DNase1L2/DNase2 triple knockout (**B**) mice were subjected to TUNEL labeling and counter-stained with the DNA-specific dye Hoechst 33258. The data are representative of at least 3 mice per genotype. Note that TUNEL-positive DNA fragments were present in the presence (**A**) and absence (**B**) of DNase1. Scales bars, 40 µm.

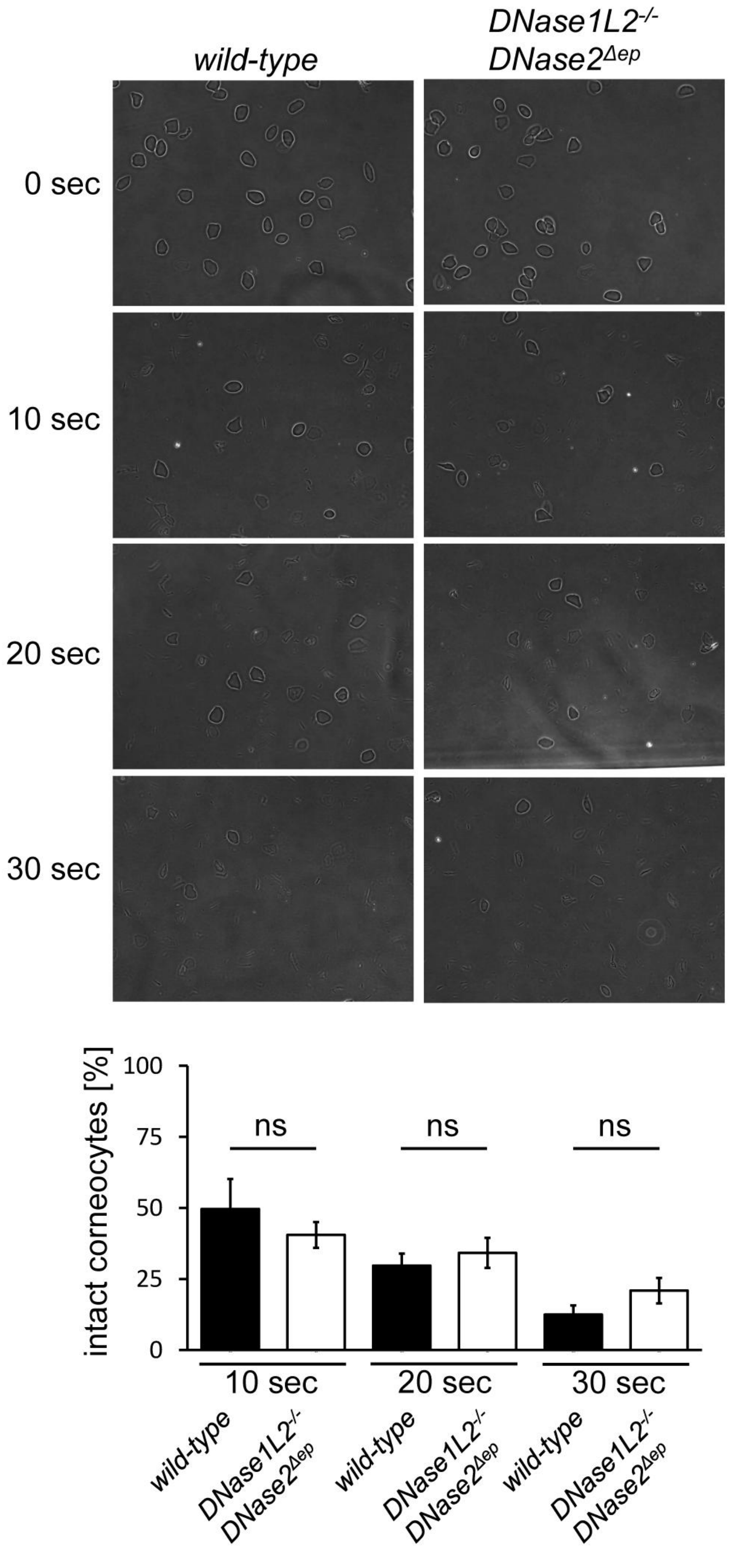

Suppl. Fig. S3. Corneocyte stress test. Corneocytes from the ears of wild-type and DNase1L2/DNase2 double knockout mice were stressed by ultrasound pulses for 0, 10, 20, and 30 seconds. Numbers of intact corneocytes were counted and the percentages relative to the starting concentrations of corneocytes were calculated. The bars indicate the means and the error bars indicate the standard deviations of experiments using samples from > 5 mice per genotype. n.s., not significant (t-test).

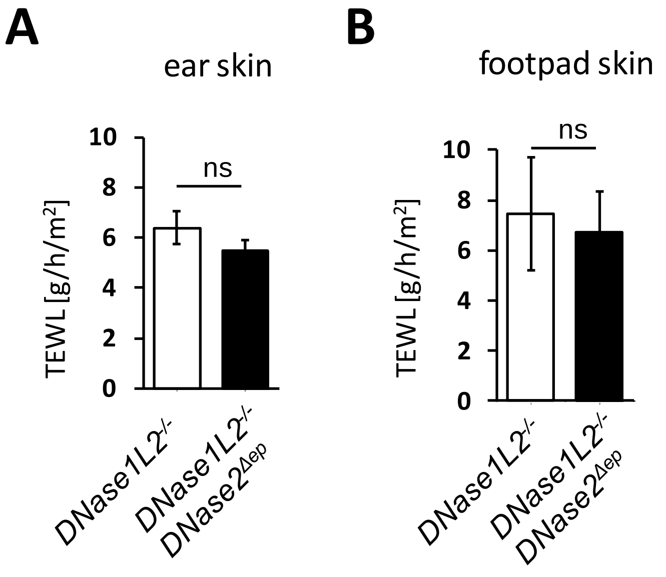

Suppl. Fig. S4. Parakeratosis in DNase1L2/DNase2 deficient mice is not associated with altered TEWL. TEWL was measured on ear skin and footpads of 3 mice per genotype. Pairs 2 mice were investigated as indicated in the individual panels. The bars show the mean of 7 measurements per body site. The error bars indicate the standard deviation. The unit of TEWL is $g/h/m^2$ as determined by measurement with a Tewameter (Courage and Khazaka).

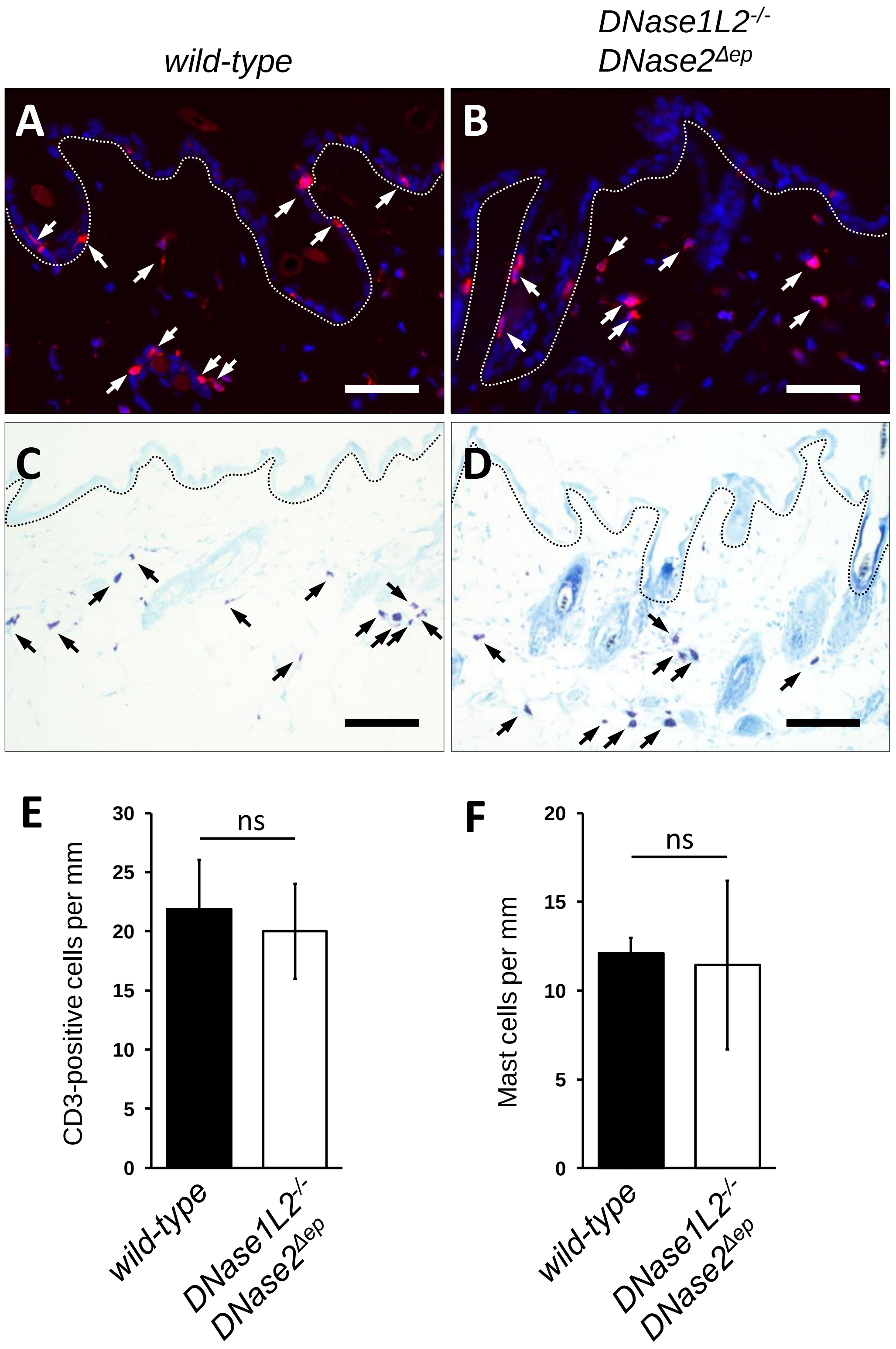

Suppl. Fig. S5. Parakeratosis in DNase1L2/DNase2 deficient mice is not associated with inflammation. Thin section of the skin on the back of DNase1L2-knockout (**A**, **C**, **E**) and DNase1L2/DNase2 double knockout (**B**, **D**, **F**) mice were immunolabeled with anti-CD3 to visualize T cells (**A**, **B**) and stained with toluidine blue to visualize mast cells (**C**, **D**). The sections were counter-stained with hematoxylin. The data are representative of at least 3 mice per genotype. Numbers of T cells (**E)** and mast cells (**F**) per mm of skin were counted and compared between the genotypes. Bars indicate means and error bars indicate standard deviations of experiments using samples from 3 mice per genotype. n.s., not significant (t-test). Scales bars, 100 µm.
